# Retrospective analysis of sexually transmitted infections among people living with HIV and pre-exposure prophylaxis users in Spain

**DOI:** 10.1186/s12879-025-12395-z

**Published:** 2025-12-23

**Authors:** María del Mar Arcos-Rueda, Luis Ramos-Ruperto, Carmen Busca, Alejandro de Gea Grela, Fernando Fernández-Hinojal, Ana Delgado Hierro, Inmaculada Quiles-Melero, Alfredo Maldonado-Barrueco, Rafael Mican, Luz Martin-Carbonero, Jose Ignacio Bernardino

**Affiliations:** 1 HIV Unit, Internal Medicine Department, Hospital Universitario La Paz-Carlos IIIIdiPAZ, Madrid, Spain; 2 Clinical Microbiology Department, Hospital Universitario La Paz-Carlos III. IdiPAZ, Madrid, Spain; 3 CIBER Enfermedades Infecciosas. Instituto de Salud Carlos III, Madrid, Spain

**Keywords:** Sexually transmitted infections, Pre-exposure prophylaxis, STI, PrEP, PWH

## Abstract

**Objective:**

This study aimed to compare the frequency and characteristics of sexually transmitted infections (STI) between people with HIV (PWH) and pre-exposure prophylaxis (PrEP) users.

**Methods:**

A retrospective analysis was conducted using nucleic acid amplification techniques (NAAT) for *Neisseria gonorrhoeae*, *Chlamydia trachomatis*, and *Mycoplasma genitalium in* rectal, urine, and pharyngeal samples, as well as serological tests for syphilis. The samples were collected and analysed as part of routine clinical care and sent to the microbiology department between Jan 1 2023, and May 31 2023. Multivariable logistic regression was performed to identify factors associated with STI diagnosis; adjusted odds ratios (aOR) and 95% confidence intervals (CI) were reported.

**Results:**

A total of 459 samples from 450 participants (277 PWH and 173 on PrEP) were included in the analysis. Overall, 144 (32%) participants tested positive for at least one STI. PrEP users had a lower median age (35 vs. 42 years; *p* < 0.01), were more frequently born in Spain (77.5% vs. 57.6%; *p* < 0.01), had a higher level of education (university 62.4% vs. 48.4%; *p* < 0.01), and had a higher prevalence of *chemsex* use (18.4% vs. 12.9%; *p* < 0.01). The prevalence of STIs was significantly higher among the PrEP users (38.2% vs. 28.2%; *p* = 0.027). However, this difference was insignificant after multivariable adjustment (aOR 1.09, 95% CI 0.6–1.7). *Neisseria gonorrhoeae* was the most common pathogen among PrEP users, while *Chlamydia trachomatis* and *Mycoplasma genitalium* were more frequent in PWH. Independent factors associated with STI diagnosis included *chemsex* use (aOR 1.9, 95% CI 1.1–3.3) and higher educational level (aOR 2.4, 95% CI 1.7–3.4).

**Conclusion:**

STIs were commonly diagnosed among PWH and PrEP users, particularly in individuals engaging in *chemsex*. The different bacterial profiles of STI between PWH and PrEP users underline the importance of continuous STI surveillance.

**Clinical trial number:**

Not applicable.

**Supplementary Information:**

The online version contains supplementary material available at 10.1186/s12879-025-12395-z.

## Background

Pre-exposure Prophylaxis (PrEP) has proven to be an effective strategy for preventing human immunodeficiency virus (HIV) infection in high-risk individuals [1–3]. Extensive scientific evidence indicates that implementation of this strategy yields a risk reduction of nearly 90% when adherence is optimal. PrEP as part of combination prevention strategies has contributed to a gradual decrease in HIV incidence in recent years, where these strategies are widely available [3–7].

In contrast to the decrease in the incidence of new HIV infections after PrEP use, diagnoses of sexually transmitted infections (STI) have experienced an upward trajectory over the past two decades, predominantly due to bacterial aetiology. Notably, the incidence of syphilis, gonorrhoea, and chlamydia has escalated, with more than 80% of cases manifesting in young males, specifically among men who have sex with men (MSM) [8–10].

In clinical trials and observational studies, including mainly MSM subjects, a notable proportion of PrEP users were diagnosed with one or more STI [11–14]. Uncertainties persist regarding whether the utilisation of PrEP is directly linked to an elevated incidence of STI [15–19], [20]. A recent systematic review and meta-analysis demonstrated that the initiation of PrEP was associated with a significant increase in the incidence of bacterial STIs among MSM [21]. However, contrasting findings have also been reported; for instance, a German cohort observed a decrease in bacterial STI incidence among PrEP users [22]. Several behavioural and testing policy strategies can influence the data. HIV and STI acquisition have the same risk factors, which are the conditions required to prescribe PrEP. Moreover, some studies have documented a significant decline in condom use among PrEP users [23]. Finally, most PrEP programs incorporate systematic testing for STI that also detect asymptomatic infections [24, 25].

Furthermore, recent publications show that among PrEP users, there are different risk profiles for acquiring STI, being more frequent in subgroups with high-risk behaviours, such as younger age, a higher number of sexual partners, unprotected anal intercourse, and the use of *chemsex* stand out [20, 23].

While many studies have explored STI trends among PrEP users, people living with HIV (PWH) also represent a population with ongoing risk of STI acquisition, especially among men who have sex with men (MSM), due to overlapping behavioural and biological factors [26]. Although antiretroviral treatment suppresses HIV replication, it does not mitigate the risk of other STIs, and PWH often undergo less systematic STI screening compared to PrEP users. A large French survey among MSM found that both PrEP use and HIV-positive status were associated with bacterial STI diagnosis in univariate analyses; however, these associations disappeared in multivariable models, where younger age, Chemsex use, and a higher number of sexual partners remained as independent predictors [27]. This suggests that the apparent link between PrEP use, HIV status, and STI acquisition may be largely explained by shared behavioural risk factors. Given that both groups are followed in similar clinical settings, a comparative analysis of STI prevalence and patterns may help to disentangle the relative contributions of behavioural and programmatic factors, and to guide more tailored prevention strategies.

Although studies comparing the frequency of STIs between PWH and PrEP users are emerging, there are still scarce data. Therefore, this study aimed to compare the frequency and characteristics of STI between PWH and PrEP users.

## Methods

### Design and population

We designed a retrospective observational study involving all individuals in the PrEP program at the internal medicine clinic (regular visits every 3 months) and PWH (regular visits every 6 months) who were tested for STI at the microbiology department between Jan 1 and May 31 2023, regardless of the presence or absence of symptoms. This analysis incorporated data from both regular visits and unexpected events (such as symptomatic STI or recent sexual partners with STI).

Screening for sexually transmitted infections was conducted, encompassing serological assessments for syphilis as well as nucleic acid amplification testing (NAAT) targeting sexually transmitted bacteria. According to national guidelines, prep users were seen every 3 months and PWH every 6 months [22]. HIV and syphilis serology was performed every 3 months in Prep users and syphilis serology every 6 months in PWH. NAAT for sexually transmitted bacteria was performed every 6 months in Prep users. PWH are tested for STI only if symptomatic, but during the study, most PWH (59.2%) were screened with NAAT every 6 months. The study included first-void urine (FVU), rectal, and pharyngeal swabs to detect *Neisseria gonorrhoeae*, *Chlamydia trachomatis*, and *Mycoplasma genitalium*, among others, using the Allplex™ 7 STI Essential Assay (Seegene^®^, Seoul, South Korea). Targets amplified with a cycle threshold (Ct) value ≤ 40 were classified as positive. *C. trachomatis.* Lymphogranuloma venereum serotype (LGV, only on rectal samples) was detected using Allplex™ Genital Ulcer Assay (Seegene^®^). Serological IgG testing for syphilis and HIV screening was performed by Atellica (Atellica™, Siemens Healthcare Diagnostics^®^, Germany).

For each clinical episode, defined as a positive result for any microorganism in the aforementioned tests, the following details were documented: the anatomical location of the isolate (rectum, pharynx, FVU), the presence or absence of associated symptoms, and macrolide resistance in cases of *M. genitalium* using Allplex™ MG & AziR assay (Seegene^®^). *N. gonorrhoeae* isolated in culture on pharyngeal or rectal swabs, and resistance to quinolones, tetracycline, and cephalosporins by gradient diffusion using MIC Test Strip™ (Liofilchem^®^, Roseto degli Abruzzi, Italy). Furthermore, each episode was explicitly designated as concurrent or non-concurrent with the diagnosis of any other STI.

### Outcomes

The primary objective of this study was to compare the prevalence of STIs between people with HIV and pre-exposure prophylaxis users. In addition, as secondary objectives, we aimed to describe STIs’ clinical characteristics and anatomical distribution, identify differences in the etiological agents involved, and assess the presence of co-infections and antimicrobial resistance. We also explored associations between STI episodes and behavioural factors, including alcohol use (defined as > 3 standard drinks per day or > 80 g on weekends), tobacco consumption (current smoker), and c*hemsex* (defined as the use of psychoactive substances such as GHB/GBL, mephedrone, or methamphetamine in a sexual context, based on patient self-report during clinical interviews).

Data were extracted from electronic medical records of the Hospital La Paz cohort of PWH and PrEP users, and the local ethics committee approved the study (CEIm Hospital Universitario La Paz, 2023.865). Due to the study’s retrospective design, a waiver for written informed consent was obtained. Confidentiality was preserved according to institutional and data protection regulations; All personal identifiers were removed, and each participant was assigned a unique study code, ensuring that no information could be traced back to individual patients. The research team had access only to de-identified datasets. The study was performed under Good Clinical Practices and the Declaration of Helsinki.

### Statistical analysis

Descriptive features of the patient population are reported as absolute numbers and percentages or as medians and interquartile ranges. Baseline characteristics were compared between PrEP users and PWH using the χ2 test, Fisher’s exact test for categorical variables, and the Mann–Whitney test for continuous variables. Differences were considered statistically significant at *p* < 0.05. Univariable logistic regression analyses were performed to identify factors associated with STI diagnosis. Variables with a p-value < 0.10 in the univariable analysis and clinically relevant factors based on previous literature were included in the multivariable logistic regression model. The final model included age, country of origin (Spanish vs. non-Spanish), education level (university vs. non-university), and *chemsex* use. Adjusted odds ratios (ORs) with 95% confidence intervals (CI) were calculated. Statistical significance was set at *p* < 0.05. Analyses were performed using the SPSS software package (version 25.0, Chicago, IL, USA).

## Results

### Baseline characteristics

The analysis included 459 episodes in 450 participants undergoing regular follow-up at our clinic during the study period, with 277 being PWH and 173 on PrEP.

The baseline characteristics of PrEP users and PWH participants are summarised in Table 1. PrEP users were younger (35 vs. 42 years; *p* < 0.01), more frequently of Spanish origin (77.5% vs. 57.6%; *p* < 0.01), had a higher level of education (university 62.4% vs. 48.4%; *p* < 0.01), and a higher prevalence of *chemsex* use (18.4% vs. 12.9%; *p* < 0.01). Most patients in both groups (98.8% and 98.2%) were cis-men, and there were two transgender women among the PrEP users and five among the PWH. All cis-men participants were MSM. Most PWH had an undetectable viral load and a CD4 + T cell count above 500 cells/mm^3^.

### **Sexually transmitted infections analysis**

Overall, 144 individuals (32%) were diagnosed with at least one STI, and 69 (47.9%) had symptoms. The frequency of STIs differed significantly between the PrEP and PWH participants, with rates of 38.2% and 28.2%, respectively (*p* = 0.027). The proportion of symptomatic STIs was slightly higher among PWH compared with PrEP users (55.1% vs. 39.4%), although this difference did not reach statistical significance (*p* = 0.08).

A total of 196 samples yielded positive results: 103 in PWH (52.5%) and 93 in PrEP users (47.5%). PWH exhibited more symptomatic episodes caused by *C. trachomatis* (65% vs. 28%, *p* = 0.026), although this was not the case for *N. gonorrhoeae* (30% vs. 27%, *p* = 0.7) or *M. genitalium* (41% vs. 29%, *p* = 0.2).

Regarding the type of microorganism involved (Table 2), *N. gonorrhoeae* was more frequently isolated among PrEP users (38 vs. 29 episodes, *p* < 0.01), whereas *C. trachomatis*, *M. genitalium*, and *Treponema pallidum* were more prevalent in PWH. These differences were not statistically significant (20 vs. 17 episodes, *p* = 0.7; 27 vs. 17 episodes, *p* = 0.29; and 16 vs. 8 episodes, *p* = 0.17).

*N. gonorrhoeae* was isolated by NAAT in 80 episodes (40.81%): 41 rectal, 30 pharyngeal swabs, and 9 FVU; in 12 episodes, it was isolated in more than one location simultaneously. Among these, 28 (35%) were cultured, with growth observed in eight (28.5%). Seven strains were resistant to quinolones (87.5%). No resistance to macrolides or cephalosporins was observed. One isolate was resistant to tetracycline.

*C. trachomatis* was detected in 44 episodes (22.4%) by NAAT: 31 rectal samples (11 LGV), 7 FVU, and 6 pharyngeal swabs; in 5 episodes, it was isolated in multiple locations simultaneously. No difference was observed between PWH and PrEP in *C. trachomatis* LGV.

Finally, *M. genitalium* was detected in 48 cases (24.5%): 25 in FVU and 23 in rectal swabs in four cases with more than one simultaneous location. Resistance to macrolides was detected in 27 (61.4%) samples. Macrolide resistance mutations are shown in Table 3.

In the univariable analysis, STI diagnosis was significantly associated with younger age, Spanish origin, *chemsex* use, and having a university-level education (Table 4). In the multivariable logistic regression model, *chemsex* use (aOR 1.9, 95% CI 1.1–3.3) and higher education level (aOR 2.4, 95% CI 1.7–3.4) remained independently associated with an increased likelihood of having an STI. Use of PrEP was not independently associated with STI diagnosis (aOR 1.09, 95% CI 0.6–1.7; *p* = 0.7).

## Discussion

The findings of our study revealed that STI diagnoses were more prevalent in PrEP users than in PWH. However, after multivariable adjustment for age, origin, *chemsex*, and education level, the use of PrEP was not significantly associated with an STI episode.

To our knowledge, this is the first study to directly compare the STI frequency and characteristics in a cohort of PWH and PrEP users under clinical care in the same healthcare facility. We found that the frequency of bacterial STI was 28.2% in PWH and 38.2% in PrEP users. These numbers are consistent with those reported in recent studies. A meta-analysis by Ebrahimi et al. [26] showed that the prevalence of STI among PWH was 30.23% (95% CI, 26.1–34.4%). A Spanish study found a two-year stable 31–35% rate of STI among PrEP users [28]. While the difference in STI between Prep users and PWH was statistically significant in the crude analysis, it did not persist after adjusting for behavioural and demographic factors. This suggests that the observed difference may be explained by underlying risk profiles rather than by using PrEP. Similar trends have been reported in other cohorts, where PrEP users often exhibit higher-risk sexual behaviours such as *chemsex* or a higher number of sexual partners [6, 18, 25]. Different studies carried out in our same setting have demonstrated that *chemsex* use is an apparent risk factor for STI [28, 29]. A more frequent STI screening in the PreP group could also explain this result. Therefore, comparisons between these groups must be interpreted cautiously, especially when screening frequency differences exist. The clinical relevance of this 10% difference is uncertain, and in the absence of a universally accepted threshold for what constitutes a meaningful difference in sexually transmitted infection rates, it may not justify differential policy decisions.

We found that *Neisseria gonorrhoeae* was the most frequently diagnosed STI among PrEP users, consistent with findings from some national studies [30], but differing from other cohorts such as SwissPrEPared, where *Chlamydia trachomatis* was more prevalent [18]. Several factors may explain this discrepancy. Firstly, *N. gonorrhoeae* has a high rate of asymptomatic infection, particularly in the pharynx and rectum, which may lead to frequent detection in settings with systematic screening protocols. Secondly, variations in local epidemiology, sexual practices, and antimicrobial resistance patterns can influence the relative distribution of pathogens across populations [12, 25]. Additionally, *gonorrhoea* may be more easily transmitted than chlamydia in some contexts, particularly through oral sex, which is less commonly protected by condom use. These nuances highlight the importance of local STI surveillance in informing targeted prevention strategies. A recent Dutch study, the AMPrEP project, demonstrated that the highest incidence of STI is concentrated in a subgroup of PrEP users characterised by younger age, higher condomless anal sex, and those involved in *chemsex* use [31]. In our multivariable analysis, *chemsex* use and higher educational level were independently associated with an increased likelihood of STI diagnosis. The association between *chemsex* and STI acquisition has been consistently documented [18, 20, 25], as the practice often involves prolonged sexual sessions, multiple partners, and reduced condom use. Interestingly, a higher level of education has also emerged as a risk factor, possibly reflecting greater access to PrEP and sexual health services, more frequent testing, or higher engagement in sexual networking environments (e.g., dating apps or saunas). This finding challenges traditional assumptions that lower education correlates with poorer health outcomes and highlights the complexity of behavioural risk patterns in MSM populations. It also underscores the need for nuanced prevention strategies beyond traditional demographic predictors. The association between higher educational level and STI diagnosis may seem counterintuitive, as education is generally linked to healthier behaviours. However, it is possible that individuals with higher education have greater access to information, healthcare resources, and preventive tools such as PrEP. In our context, several studies have described chemsex users as predominantly individuals with university-level education [32–34], but none have found educational level to be an independent predictor of chemsex engagement.

PWH had a significantly higher proportion of symptomatic *C. trachomatis* infections than PrEP users. The diagnostic approach may partially explain this difference: while PrEP users undergo systematic screening every six months -allowing detection of both symptomatic and asymptomatic infections—, STI testing in PWH is often symptom-driven unless they are part of a structured screening program. Consequently, asymptomatic infections in PWH are more likely to go undetected, and the infections that are identified tend to be symptomatic. Nevertheless, a substantial proportion of PWH subjects included in this study (59.2%) were part of a specific program of systematic STI screening every 6 months. This distinction highlights a critical methodological consideration when comparing symptomatology between groups and suggests that differences in clinical presentation might reflect screening strategies rather than biological differences in disease expression.

This study has several limitations. First, although its retrospective design limits causal inference, the main constraint was the short observation period of five months, which precluded the assessment of temporal trends. Second, the difference in screening frequency between groups may have led to differential detection of asymptomatic infections. Third, we did not collect data on key behavioural variables such as condom use, number of sexual partners, or specific sexual practices, which may have influenced STI risk. Additionally, the short study period did not help assess possible seasonal variations in STI prevalence. Finally, the study population was composed predominantly of MSM attending a single urban clinic, limiting the generalizability of our findings to other settings or populations.

Future research should include longer-term prospective studies incorporating behavioural data and repeated measures to better characterise STI dynamics among PrEP users and PWH. From a public health perspective, interventions aimed at improving access to regular STI testing are warranted. Expanding the availability of self-sampling or community-based STI screening could reduce diagnostic gaps and improve early detection. Additionally, integrating behavioural and educational interventions tailored to high-risk subgroups, including chemsex users, may enhance prevention efforts beyond traditional biomedical strategies.

## Conclusion

The diagnosis of STIs was common in PrEP users and young PWH, with notably higher prevalence among individuals engaging in *chemsex* and those with higher educational levels. Our findings reveal distinct STI profiles between these two populations, highlighting the need for tailored prevention strategies and ongoing epidemiological surveillance.

**Table 1:** Baseline demographic and clinical characteristics

	PWH (*n* = 277)	PrEP (*n* = 173)	*p*
Age, median (IQR)	42 (35–52)	35 (30–40)	< 0.0001
Gender (cis male) n(%)	273 (98.2)	171(98.8)	
Sexual orientation (MSM) n (%)	277 (100)	173 (100)	
Origin (Spanish born) n(%)	160(57.6)	134 (77.5)	< 0.0001
Education level n(%)			< 0.0001
Primary	49 (17.6)	28 (16.2)	
Secondary	87 (31.4)	22 (12.7)	
University	134 (48.4)	108 (62.4)	
Unkown	7 (2.6)	15 (8.7)	
Alcohol n(%)	112(42.4)	83(48.8)	
Current smoking n(%)	102(38.2)	45(26.3)	0.01
*Chemsex* use n(%)	34(12.9)	26.9(18.4)	< 0.0001
STI episodes n(%)	78 (28.2)	66 (38.2)	0.027
Symptomatic STI n(%)	43 (55.1)	26 (39.4)	0.08
**PWH variables**			
HIV-RNA (< 50cp/mL) n(%)	255 (92%)		
T-CD4 + cell count (cells/mm^3^) n(%)	694 (549–874)		
**PrEP modality**			
Daily n(%)		140 (80)	
On demand n(%)		33 (20)	

**Table 2:** Descriptive analysis

	PWH (*n* = 103)	PrEP (*n* = 93)	*p*
***Chlamydia trachomatis*** n, (%)	23 (22.3)	21 (22.5)	0.7
Pharyngeal n	4	2	0.6
Urine n	2	5	0.08
Rectal n	17	14	0.09
Symptomatic n, (%)	15 (65.2)	6 (65.2)	0.026
*Lymphogranuloma venereum (rectal samples screening)* n	8	3	0.1
***Neisseria gonorrhoeae*** n, (%)	33 (32)	47 (50.5)	0.006
Pharyngeal n	12	18	0.1
Urine n	3	6	0.1
Rectal n	18	23	0.3
Symptomatic n, (%)	10 (30.3)	13 (27.6)	0.7
Culture collected n	13	15	0.8
Culture growth n	4	4	0.7
***Mycoplasma genitalium*** n, (%)	31 (30)	17 (18.3)	0.29
Urine n	13	12	0.38
Rectal n	18	5	0.019
Symptomatic n	13	5	0.2
Macrolid resistance n	17	10	0.7
***Treponema pallidum*** n, (%)	16 (15.5)	8 (8.6)	0.17
First episode n	3	4	0.11

**Table 3:** Macrolides resistance mutations

Mutations	*N* = 27
A2058T	7
A2058G	3
A2059G	10
Inhibid NAAT	1
No data	6

**Table 4:** Univariable and multivariable logistic regression analyses of factors associate with STI diagnosis

	OR (95% CI) Univariable	*p*-value Univariable	aOR (95% CI) Multivariable	*p*-value Multivariable
PrEP use	1.6 (1.1–2.4)	0.027	1.09 (0.6–1.7)	0.7
*Chemsex*	2.5 (1.6–3.9)	< 0.001	1.9 (1.1–3.3)	0.024
Higher education	2.8 (1.9–4.1)	< 0.001	2.4 (1.7–3.4)	< 0.001
Spanish origin	1.3 (0.9–2.0)	0.12	—	—
Age (younger)	1.4 (0.9–2.2)	0.11	—	—

## Supplementary Information

Below is the link to the electronic supplementary material.

Supplementary Material 1

## Data Availability

The data supporting this study’s findings are not openly available due to reasons of sensitivity and are available from the corresponding author upon reasonable request.

## Abbreviations

HIV: Human immunodeficiency virus
PWH: People with HIV
PrEP: Pre-exposure prophylaxis
STI: Sexually transmitted infections
MSM: Men who have sex with men
NAAT: Nucleic acid amplification techniques
IQR: Interquartile range
