# Correction: Retrospective analysis of sexually transmitted infections among people living with HIV and pre-exposure prophylaxis users in Spain

**DOI:** 10.1186/s12879-026-13136-6

**Published:** 2026-03-30

**Authors:** María del Mar Arcos-Rueda, Luis Ramos-Ruperto, Carmen Busca, Alejandro de Gea Grela, Fernando Fernández-Hinojal, Ana Delgado Hierro, Inmaculada Quiles-Melero, Alfredo Maldonado-Barrueco, Rafael Mican, Luz Martin-Carbonero, Jose Ignacio Bernardino

**Affiliations:** 1 HIV Unit, Internal Medicine Department, Hospital Universitario La Paz-Carlos IIIIdiPAZ, Madrid, Spain; 2 Clinical Microbiology Department, Hospital Universitario La Paz-Carlos III. IdiPAZ, Madrid, Spain; 3 CIBER Enfermedades Infecciosas. Instituto de Salud Carlos III, Madrid, Spain

**Author Correction: BMC Infectious Diseases (2025) 26:167**

10.1186/s12879-025-12395-z

In the original version of this article, the given and family names of the following authors were incorrectly structured:

Arcos-Rueda María del Mar, Ramos-Ruperto Luis, Busca Carmen, de Gea Grela Alejandro, Fernández-Hinojal Fernando, Delgado Hierro Ana, Quiles-Melero Inmaculada, Maldonado-Barrueco Alfredo, Mican Rafael, Martin-Carbonero Luz and Bernardino Jose Ignacio

The names were displayed correctly in all published versions.

The correct structure should be as follows:

María del Mar Arcos-Rueda (given name: María del Mar; surname: Arcos-Rueda),

Luis Ramos-Ruperto (given name: Luis; surname: Ramos-Ruperto),

Carmen Busca (given name: Carmen; surname: Busca),

Alejandro de Gea Grela (given name: Alejandro; surname: de Gea Grela),

Fernando Fernández-Hinojal (given name: Fernando; surname: Fernández-Hinojal),

Ana Delgado Hierro (given name: Ana; surname: Delgado Hierro),

Inmaculada Quiles-Melero (given name: Inmaculada; surname: Quiles-Melero),

Alfredo Maldonado-Barrueco (given name: Alfredo; surname: Maldonado-Barrueco),

Rafael Mican (given name: Rafael; surname: Mican),

Luz Martin-Carbonero (given name: Luz; surname: Martin-Carbonero), and

Jose Ignacio Bernardino (given name: Jose Ignacio; surname: Bernardino).]

The original article has been corrected.

