# Supplementary material for: Retrospective analysis of sexually transmitted infections among people living with HIV and pre-exposure prophylaxis users in Spain
Source: BMC Infect Dis. 2025 Dec 23;26:167. doi: 10.1186/s12879-025-12395-z (PMC12837009; doi:10.1186/s12879-025-12395-z)
Supplement: Supplementary file 1 — Supplementary Material 1 [file 12879_2025_12395_MOESM1_ESM.docx]

# STROBE Checklist for Retrospective Cohort Study

Title of manuscript: Retrospective Analysis of Sexually Transmitted Infections among People Living with HIV and Pre-exposure Prophylaxis Users in Spain

| Item No | Recommendation | Location in manuscript |
| --- | --- | --- |
| 1(a) | Indicate the study’s design in the title or abstract | Title (line 1-3), Abstract (line 39) |
| 1(b) | Provide an informative and balanced summary | Abstract (lines 31–63) |
| 2 | Scientific background and rationale | Background (lines 65–103) |
| 3 | Objectives and hypotheses | End of Background (lines 107–108), Methods- Outcomes (lines 150-159) |
| 4 | Key elements of study design | Methods – Design and population (lines 114-119) |
| 5 | Setting, locations, relevant dates | Methods – Design and population (lines 121–146) |
| 6(a) | Eligibility, selection and follow-up | Methods – Design and population (lines 114-119, 138-146) |
| 6(b) | Matching criteria (if any) | N/A – not a matched study |
| 7 | Define outcomes, exposures, confounders | Methods – Outcomes (lines 150-159) |
| 8 | Data sources and measurement | Methods – Design and population (lines 128–146), outcomes (lines 161-166) |
| 9 | Potential sources of bias | Discussion – Limitations (lines 316–324) |
| 10 | How study size was determined | N/A |
| 11 | Handling of quantitative variables | Methods – Statistical analysis (lines 168 - 181) |
| 12(a) | Statistical methods incl. confounding | Methods – Statistical analysis (lines 168 - 181) |
| 12(b) | Subgroup and interaction methods | N/A – not applicable |
| 12(c) | Missing data | Discussion – Limitations (lines 316–324) |
| 12(d) | Loss to follow-up | N/A – retrospective study |
| 12(e) | Sensitivity analyses | N/A – not performed |
| 13(a) | Individuals at each stage | Results – Baseline characteristics (lines 186-187) |
| 13(b) | Reasons for non-participation | N/A |
| 13(c) | Flow diagram | N/A – retrospective, not included |
| 14(a) | Participant characteristics | Results – Table 1 and lines 190-196 |
| 14(b) | Missing data for each variable | Discussion – Limitations (lines 316–324) |
| 14(c) | Follow-up time | Methods-(Line 117) |
| 15 | Outcome events or summary measures | Results – STI analysis (lines 200-205) |
| 16(a) | Estimates and confidence intervals | Results – Tables 2 and 4 (lines 201-237) |
| 16(b) | Variable categories | Methods – Statistical analysis (lines 168 - 181) |
| 16(c) | Relative to absolute risk | N/A – |
| 17 | Other analyses | Results – Logistic regression (lines 239-244) |
| 18 | Key results | Discussion – Opening (lines 247 - 249) |
| 19 | Study limitations | Discussion – Limitations (lines 316–324) |
| 20 | Interpretation of results | Discussion – Full section (lines 247 – 314) |
| 21 | Generalisability | Discussion – Limitations (lines 316–324) |
| 22 | Funding and role of funders | Declarations – Funding (line 157) |
